# Jones-matrix imaging based on two-photon interference

**DOI:** 10.1515/nanoph-2022-0499

**Published:** 2022-10-24

**Authors:** Tsz Kit Yung, Hong Liang, Jiawei Xi, Wing Yim Tam, Jensen Li

**Affiliations:** Department of Physics, The Hong Kong University of Science and Technology, Clear Water Bay, Hong Kong, China

**Keywords:** Jones-matrix imaging, quantum imaging, quantum metasurfaces, two-photon interference

## Abstract

Two-photon interference is an important effect that is tightly related to the quantum nature of light. Recently, it has been shown that the photon bunching from the Hong–Ou–Mandel (HOM) effect can be used for quantum imaging in which sample properties (reflection/transmission amplitude, phase delay, or polarization) can be characterized at the pixel-by-pixel level. In this work, we perform Jones matrix imaging for an unknown object based on two-photon interference. By using a reference metasurface with panels of known polarization responses in pairwise coincidence measurements, the object’s polarization responses at each pixel can be retrieved from the dependence of the coincidence visibility as a function of the reference polarization. The post-selection of coincidence images with specific reference polarization in our approach eliminates the need in switching the incident polarization and thus parallelized optical measurements for Jones matrix characterization. The parallelization in preparing input states, prevalent in any quantum algorithms, is an advantage of adopting two-photon interference in Jones matrix imaging. We believe our work points to the usage of metasurfaces in biological and medical imaging in the quantum optical regime.

## 1 Introduction

Nanostructured metasurfaces allow manipulation of the different degrees of freedom (DOF) of light with fine resolution [1–3]. Due to their promising capability in bringing down the size, weight, and power of a device, many efforts have been made to adopt metasurfaces for imaging applications with various optical properties, for example, polarization [4–9], phase [10–14], wavelength [15–19], and view depth [20–22]. Compared to conventional cameras that simply project a 3D object into 2D intensity profile, the integration of metasurface into detectors/camera permits the construction of a complex point spread function that is necessary for multidimensional light field imaging [21]. The complex information in the light field can then be used in many applications such as autonomous driving for depth sensing, target recognition and material composition identification for spectral imaging, image contrast enhancement, stress detection for polarimetric imaging, and more recently on implementing neural networks [23, 24] and brain-computer interface [25]. In quantum optics, the manipulation of individual photons, in terms of quantum interference and entanglement, has been a primary interest in the field [26]. The use of metasurfaces in constructing quantum optical sources allows for generating complex entangled states [27–29], while on the detection side metasurfaces offer applications like one-shot state tomography [30]. Among these applications, two-photon interference is an important effect involving two-photon states. In this case, two indistinguishable photons can have two different quantum processes undergoing either destructive or constructive interference to trigger a two-photon coincidence count at two detectors. The resulting interference in coincidence events among two output ports has been observed for spatial and polarization modes, conventionally with a beam splitter, now with metasurfaces in more general scenarios [31–34].

For the application of quantum imaging, coincidence events are measured in a ghost-imaging setting between the signal (imaging) photons and the heralding photons in deciding whether the detected photons on the image should be recorded or not. For example, two independent images can be superimposed through quantum entanglement and obtained respectively by projecting heralding photons onto different states, with applications on quantum edge detection and imaging with entangled photons [35, 36]. In the latest development, it has been shown that two-photon interference and the resulting bunched photon pairs can be used directly for quantum imaging, which either serves as an input light source to enhance the sensitivity of the imaging setup [37] or as a mechanism to sense the change in reflection/transmission amplitude [38] and phase delay [39, 40] at different locations of the object to be imaged. Note that in these imaging schemes, detection of second-order coherence *g*_2_ (coincidence count at photon level) directly reveals the effect of two-photon interference. The effect shows up in the form of intensity correlations rather than intensity in classical interference. It can be used to enhance the signal-to-noise ratio in imaging when an entangle-photon source is used [41].

For an object with arbitrary polarization properties, its behavior can be completely described by a complex 2 × 2 Jones matrix that contains 8 unknown real parameters. Conventionally, to generate a system of equations that can be inverted to determine the parameters of the Jones matrix, a series of intensity measurements are required by choosing different combinations of the incident and analyzing polarization states. Excluding the global phase of the Jones matrix, the minimal required number of intensity measurements is 10 with one measurement for each amplitude and two measurements for each relative phase difference [42–44]. Normally, to reduce the error in the retrieved phase difference, over-complete measurements are desired with more measurements than the minimal requirement. As the quantitative measurement of the Jones matrix elements is crucial for the study of light polarization in many applications, significant efforts have been made to simplify the classical approach in Jones matrix characterization with examples including vectorial Fourier ptychography [45, 46] and the method of Fourier space sharing [47, 48]. For the former example, the required number of combinations of the incident and analyzing polarization states is reduced by scanning the incident angle for each configuration. While in the latter example, the contribution from different Jones matrix elements can be isolated and retrieved independently using the concept of Fourier space sharing. In either case, the characterization of the Jones matrix is simplified through post-processing and parallelization of the experimental configurations.

**Figure 1:**
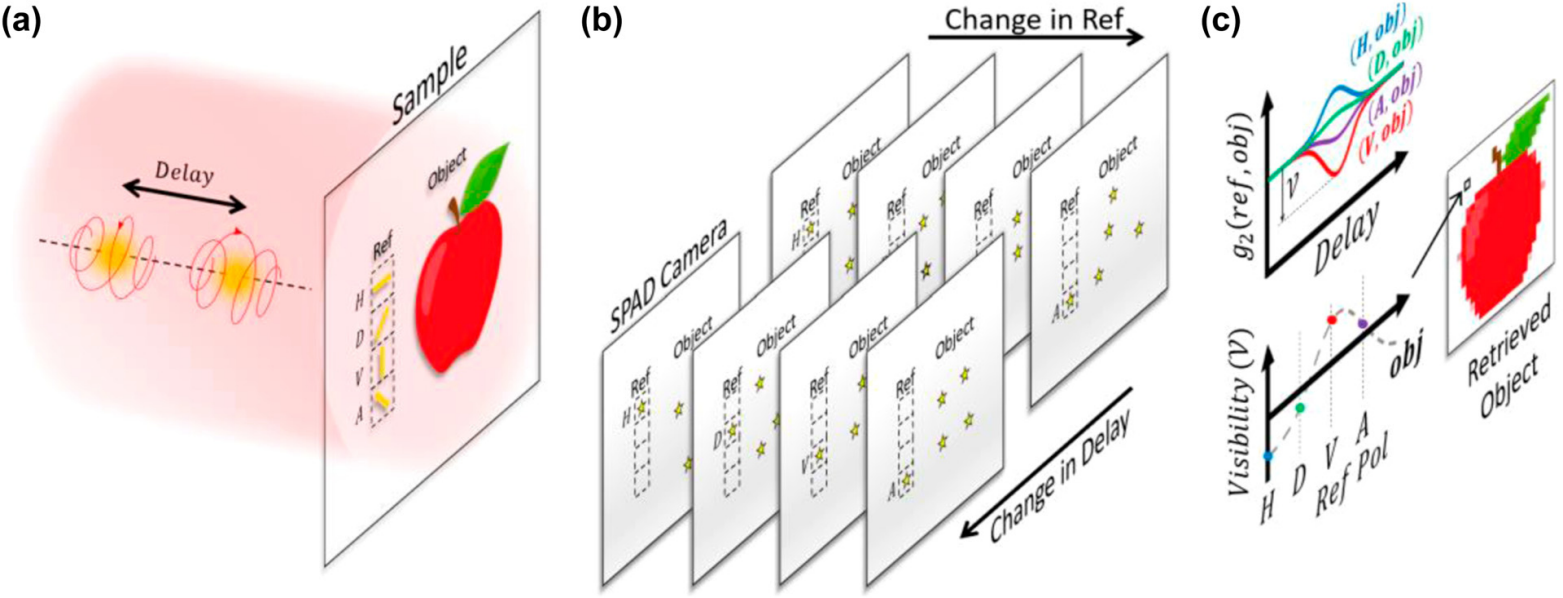
Coincidence imaging scheme to image Jones matrix elements. (a) A photon pair with orthogonal circular polarizations shining on the sample, which is imaged by an SPAD camera on the transmitted side. The sample has an object with unknown polarization responses and a metasurface (labeled as Ref) providing known polarization references (e.g. “H”, “D”, “V”, “A”) for pairwise second-order coherence *g*_2_ measurements. (b) A stack of coincidence images with respect to different reference polarizations and with different time delays between the two incident photons. (c) HOM visibility V of each *g*_2_ curve between any reference panels and an object pixel is obtained and processed to extract the Jones matrix elements, which are further assembled as the image of the object.

In this work, we have performed Jones matrix imaging for an unknown object based on two-photon interference. Figure 1 shows the schematic of the experiment. The sample is divided into two regions: an object (the “apple”) with unknown transmission polarization responses and a reference metasurface region with nano-structures of known polarization responses. For illustration, Figure 1(a) shows 4 reference panels, labeled as “H”, “D”, “V”, and “A”, passing through different chosen polarizations. When two orthogonal circularly polarized photons are shined onto the whole sample area, a series of raw images can be recorded at individual time frames by a single-photon avalanche diodes (SPAD) camera. By using an SPAD camera, it becomes possible to probe coincidence events between any two locations (pixels) within the image in a high throughput manner. The SPAD camera is now envisioned to be very useful that metasurfaces can process information in parallel and the SPAD camera can record also in parallel in the quantum regime [49]. Each pixel of a raw image has a binary value indicating whether a photon is registered or not. The whole imaging process is then repeated for different time delays between the two photons. From the raw images, a coincidence image with respect to a particular reference panel refers to an array of the number of coincidence events with one photon recorded through any of the object pixels while another is recorded through the reference panel at the same time frame. In such a way, a stack of 4D coincidence images: two spatial degrees of freedom (DOF) plus another two on the choice of the reference polarization and tunable delay is formed, as shown in Figure 1(b). Then, for a particular object pixel, we can plot the second-order coherence *g*_2_, as a curve against the tunable time delay for the different reference polarizations. A dip or a peak on such a curve is contributed by the destructive (HOM) or constructive two-photon interference. A series of *g*_2_-visibilities ( V ) of these curves are then finally obtained against the different reference panels/polarizations, as shown in Figure 1(c). These data are then processed to retrieve the Jones matrix elements of each object pixel. To have a wide range of responses to the object to demonstrate the full capability of our coincidence imaging approach, we choose to construct the object using arrays of nanoslots with variable slot lengths, orientations, and sub-lattice structures. The Jones matrix, up to 3 DOFs, can then be determined and coded using an HSB color scheme as the final image shows. We note that in the experiments here, we use weak coherent states with randomized phases as the incident source. Such an approach, instead of using photon pair, e.g. from an entangled photon source, poses a lower/upper bound of 0.5/1.5 to the *g*_2_ values [50–52] while the principle and mechanism of coincidence imaging remain the same. Despite this tradeoff, the weak coherent states provide a substantially higher incident power in exploiting quantum interference which is particularly useful in compensating losses due to transmission, especially in long ranges [53, 54], and reducing the camera’s exposure time [40].

## 2 Coincidence imaging scheme

Our imaging scheme starts with two incident pulses having complex coherent state amplitudes *α*_L_ for the left-handed circular polarization (LCP) and *α*_R_ for the right-handed circular polarization (RCP) arriving on the sample at the same time. The polarization basis is selected to exploit the Pancharatnam–Berry phase with the reference panels (polarizers) which greatly simplifies the equations in this section. The sample is then imaged by the SPAD camera for different final analyzing polarization selected by a quarter wave plate and a polarizer before the camera. In terms of *α*_L_ and *α*_R_, the output coherent amplitudes arriving at any pixels on the camera can be written as

(1) $$\begin{array}{c} \alpha_{i}=t_{i\text{L}}\alpha_{\text{L}}+t_{i\text{R}}\alpha_{\text{R}},\\ \alpha_{j}=t_{j\text{L}}\alpha_{\text{L}}+t_{j\text{R}}\alpha_{\text{R}}, \end{array}$$

where an index *i* (*j*) is used to iterate pixels that can be traced back to the reference panels (object) while the second index, “L” or “R”, of all the complex transfer amplitudes indicates either LCP or RCP incidence. By selecting all time frames that one photon falls on a specific reference panel while another on any pixels of the object area, a coincidence image is formed with respect to the passing polarization of such reference panel. In terms of the Jones matrix on circular polarization basis, a reference pixel is defined by

(2) $$\left(\begin{array}{cc} t_{\text{LL}}^{\left(i\right)} & t_{\text{LR}}^{\left(i\right)}\\ t_{\text{RL}}^{\left(i\right)} & t_{\text{RR}}^{\left(i\right)} \end{array}\right)=t_{\text{ref}}\left(\begin{array}{cc} 1 & {\text{e}}^{-2\text{i}\theta_{i}}\\ {\text{e}}^{2\text{i}\theta_{i}} & 1 \end{array}\right),$$

ideally as a polarizer where *θ*_*i*_ is the angle of the passing axis and *t*_ref_ is the overall complex transmission amplitude of the reference pixel. Without losing generality, we consider the final analyzer in probing LCP, we have $t_{i\text{L}}=t_{\text{LL}}^{\left(i\right)}=t_{\text{ref}}$ , $t_{i\text{R}}=t_{\text{LR}}^{\left(i\right)}=t_{\text{ref}}{\text{e}}^{-2\text{i}\theta_{i}}$ for a reference pixel. For an object pixel with an unknown Jones matrix $\left\{\left\{t_{\text{LL}}^{\left(j\right)},t_{\text{LR}}^{\left(j\right)}\right\},\left\{t_{\text{RL}}^{\left(j\right)},t_{\text{RR}}^{\left(j\right)}\right\}\right\}$ , we have similarly $t_{j\text{L}}=t_{\text{LL}}^{\left(j\right)}$ and $t_{j\text{R}}=t_{\text{LR}}^{\left(j\right)}$ with the LCP analyzer. Conventionally, we have to perform experiments of configurations with different combinations of *α*_L_ and *α*_R_ to probe $t_{\text{LL}}^{\left(j\right)}$ and $t_{\text{LR}}^{\left(j\right)}$ through classical interference [42–44]. However, in the current context of two-photon interference, we keep $\left|\alpha_{\text{L}}\right|=\left|\alpha_{\text{R}}\right|$ fixed with phase randomization between the two coherent amplitudes and their parallelization without the need to use different configurations is introduced in the following.

Considering the photon statistics directly from the output coherent states *α*_*i*_ and *α*_*j*_. Assume all the camera’s detectors have the same detection efficiency *η* and it does not distinguish photon number (i.e. only differentiate no photon and has photon in the same frame), the coincidence count rate *P*_*ij*_ between any pairs of a reference pixel and an object pixel is governed by [34]

(3a) $$\begin{array}{ll} P_{ij} & =\sum_{N=1}^{\infty }\sum_{M=1}^{\infty }{\left|<N_{i}M_{j}|\alpha_{i}\alpha_{j}>\right|}^{2}\left(1-{\left(1-\eta \right)}^{N}\right)\left(1-{\left(1-\eta \right)}^{M}\right). \end{array}$$

$\left|N_{i}\right\rangle$ and $\left|M_{j}\right\rangle$ are the Fock state denoting *N* and *M* photons at the pixel *i* and *j* and $\left|\alpha_{i}\alpha_{j}\right\rangle$ represents the coherent state with the corresponding complex amplitudes. From Eq. (1), when the detected number of photons per pixel per time frame is much less than 1, Eq. (3a) reduces to

(3b) $$P_{ij}≅{\left(P_{\text{ref}}^{\left(b\right)}\right)}^{2}\;\left(\frac{{\left|t_{j\text{L}}\right|}^{2}+{\left|t_{j\text{R}}\right|}^{2}}{{\left|t_{i\text{L}}\right|}^{2}+{\left|t_{i\text{R}}\right|}^{2}}+\frac{2Re\left(t_{i\text{L}}t_{i\text{R}}^{*}t_{j\text{L}}^{*}t_{j\text{R}}\right)}{{\left({\left|t_{i\text{L}}\right|}^{2}+{\left|t_{i\text{R}}\right|}^{2}\right)}^{2}}\right),$$

where $P_{\text{ref}}^{\left(b\right)}$ is the constant ballistic single photon count rate for any reference pixels and can be directly measured in the case of a large optical delay between the two pulses. The first term within the bracket of Eq. (3b) is the normalized ballistic contribution while the second term represents the scaled (by the ballistic count rate) two-photon interference between the situation that the LCP (RCP) photon falls on the reference (object) pixel through *t*_*i*L_ (*t*_*j*R_) and another situation where the LCP (RCP) photon falls on the object (reference) pixel through *t*_*j*L_ (*t*_*i*R_). Therefore, by choosing different combinations of *θ*_*i*_, *t*_*j*L_, and *t*_*j*R_ (equivalently $t_{\text{LL}}^{\left(j\right)}$ and $t_{\text{LR}}^{\left(j\right)}$ in the current case of LCP final analyzer) can be probed using the term $Re\left(t_{i\text{L}}t_{i\text{R}}^{*}t_{j\text{L}}^{*}t_{j\text{R}}\right)$ . Essentially, it is similar to probing in classical interference except that these different cases of *θ*_*i*_ can now be done by choosing different reference panels (the “H”, “D”, “V”, and “A” in Figure 1) as a post-selection, i.e. the conventional serial experiments of varying *θ*_*i*_ are now parallelized. The parallelization in preparing input states, prevalent in any quantum algorithms, is an advantage of adopting two-photon interference in Jones matrix imaging.

To evaluate the proposed imaging scheme, an object with enough complexity in the polarization response is required and is assembled here using plasmonic nano-slots in various slot lengths, orientations, and sub-lattice structures. The Jones matrix of an object pixel under consideration here is most generally defined as

(4) $$\left(\begin{array}{cc} t_{\text{LL}}^{\left(j\right)} & t_{\text{LR}}^{\left(j\right)}\\ t_{\text{RL}}^{\left(j\right)} & t_{\text{RR}}^{\left(j\right)} \end{array}\right)=t_{j}\left(\begin{array}{cc} 1 & \cos\theta_{-}^{\left(j\right)}{\text{e}}^{-\text{i}\theta_{+}^{\left(j\right)}}\\ \cos\theta_{-}^{\left(j\right)}{\text{e}}^{\text{i}\theta_{+}^{\left(j\right)}} & 1 \end{array}\right),$$

where *t*_*j*_ is the overall complex transmission amplitude of the object pixel *j*, $\theta_{+}^{\left(j\right)}$ (from 0 to 360°) and $\theta_{-}^{\left(j\right)}$ (between 0 and 90°) define the off-diagonal elements. The connection of Eq. (4) to the geometry of plasmonic nanoslots will be given later. Eq. (4) will be enough to have any complex ratio between the $t_{\text{LL}}^{\left(j\right)}$ and $t_{\text{LR}}^{\left(j\right)}$ as a demonstration for probing them using a final LCP analyzer. For more general Jones matrices, one can repeat the experiment for at most two more final analyzing polarizations to probe all the elements. By substituting Eq. (2) and (4) into Eq. (3b), we obtain

(5) $$\begin{array}{ll} P_{ij}\left.\left(\theta_{i}\right)\right. & ≅\frac{1}{2}{\left(P_{\text{ref}}^{\left(b\right)}\right)}^{2}{\left|\frac{t_{j}}{t_{\text{ref}}}\right|}^{2}(1+{\text{cos}}^{2}\theta_{-}^{\left(j\right)}\\ & \left.\;+\cos\theta_{-}^{\left(j\right)}\cos\left(2\theta_{i}-\theta_{+}^{\left(j\right)}\right)\right). \end{array}$$

*P*_*ij*_ as a function of *θ*_*i*_ can now be obtained from the experiment with *θ*_*i*_ being the different passing axis angles of the reference panels. $\theta_{+}^{\left(j\right)},\theta_{-}^{\left(j\right)}$ and ${\left|t_{j}\right|}^{2}$ can then be extracted from such a function. Note that in the experiment one needs to scan the optical delay between the two incident pulses to maximize the signal of two-photon interference. The coincidence signal $g_{2}=P_{ij}/\left(P_{\text{ref}}^{\left(b\right)}P_{j}^{\left(b\right)}\right)$ when plotted against the optical delay will exhibit a curve as shown in Figure 1(c) with a dip or a peak at zero optical delays such that the visibility $V_{ij}$ (defined as the value of 1 − *g*_2_ at the dip or peak) is related to Eq. (5) through

(6) $$V_{ij}\left(\theta_{i}\right)≜1-\frac{P_{ij}}{P_{\text{ref}}^{\left(b\right)}P_{j}^{\left(b\right)}}≅-\frac{\cos\theta_{-}^{\left(j\right)}}{1+\cos^{2}\theta_{-}^{\left(j\right)}}\cos\left(2\theta_{i}-\theta_{+}^{\left(j\right)}\right),$$

with the ballistic single photon count rate for the object pixel written as

(7) $$P_{j}^{\left(b\right)}≅\frac{1}{2}P_{\text{ref}}^{\left(b\right)}{\left|t_{j}/t_{\text{ref}}\right|}^{2}\left(1+\cos^{2}\theta_{-}^{\left(j\right)}\right).$$

Then, the amplitude and the location of interference in the experimental visibility curve in Eq. (6) against θ_*i*_ can be used to extract $\theta_{-}^{\left(j\right)}$ and $\theta_{+}^{\left(j\right)}$ while ${\left|t_{j}/t_{\text{ref}}\right|}^{2}$ is experimentally obtained from Eq. (7). In our experiment, ${\left|t_{j}/t_{\text{ref}}\right|}^{2}$ can also be extracted directly from next order contribution of $P_{\text{ref}}^{\left(b\right)}$ towards the visibility formula:

(8) $$V_{ij}\left(\theta_{i}\right)≅\beta +\gamma \cos\left(2\theta_{i}-\theta_{+}^{\left(j\right)}\right),$$

where

(9a) $$\beta ≅\frac{1}{4}P_{\text{ref}}^{\left(b\right)}\left(1+\frac{2\cos^{2}\theta_{-}^{\left(j\right)}}{1+\cos^{2}\theta_{-}^{\left(j\right)}}{\left|t_{j}/t_{\text{ref}}\right|}^{2}\right)$$

and

(9b) $$\begin{array}{ll} \gamma & ≅\left(1-\frac{1}{4}P_{\text{ref}}^{\left(b\right)}\left(2+\left(1+\cos^{2}\theta_{-}^{\left(j\right)}\right){\left|t_{j}/t_{\text{ref}}\right|}^{2}\right)\right)\\ & \;\times \frac{-\cos\theta_{-}^{\left(j\right)}}{1+\cos^{2}\theta_{-}^{\left(j\right)}}. \end{array}$$

## 3 Results and discussion

### 3.1 Sample and setup characterization

**Figure 2:**
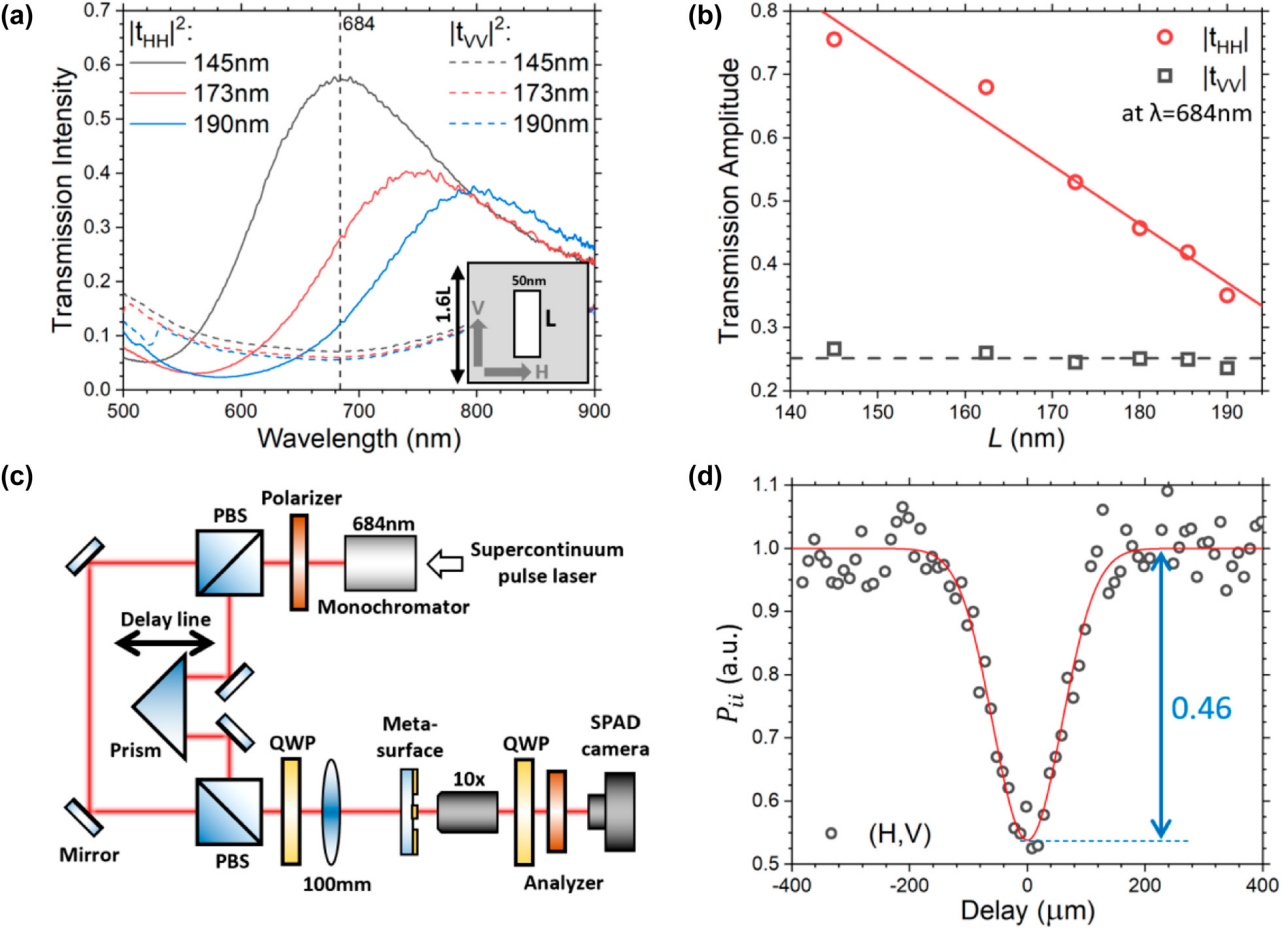
Design parameters of the metasurface and the experimental setup. (a) The measured spectrum of the vertically orientated periodic nanoslot array with slot length *L* and fixed-width at 50 nm. Inset is the unit cell (1.6*L* × 1.6*L*) of the nanoslot array. For *L* = 145 nm, the nano-slot array acts almost like a horizontal polarizer such that the transmission ratio (*T*_HH_/*T*_VV_) is maximized at 684 nm which is chosen as the working wavelength for our coincidence imaging. (b) Transmission amplitude $\left|t\right|$ at wavelength 684 nm of the nano-slot array as a function of *L*. The red solid line is a linear fit to the horizontal polarization’s data (red circles) while the black dashed line gives the mean value of the vertical polarization’s data (black open squares). The red line provides the amplitude dependence on *L* for the design of our metasurface. (c) Experimental setup for the coincidence imaging. Two orthogonal circularly polarized (RCP & LCP) pulses are prepared at 684 nm and focused onto the object and also the reference metasurface by a 100 mm lens. The transmitted light is collected by a 10× objective and projected to LCP, after passing through a quarter waveplate and a polarizer, before being captured by an SPAD camera. (d) Normalized coincidence count between reference’s horizontal (H) and vertical polarizations (V), i.e. *P*_*ii′*_ versus delay. The solid red curve is a Gaussian fit to the data.

Figure 2(a) shows the measured co-linear polarization transmission spectrum of a periodic vertically orientated nano-slot array (inset, *θ*_*i*_ = 0° for the passing axis) for different slot lengths. The nano-slot array is fabricated on a 50 nm thick silver film on a glass substrate by a focused ion beam (FIB) technique with a periodicity *p* scaled with the slot length *L* (*p* = 1.6*L* kept fixed). This ratio is selected to avoid overlapping of nano-slots for long slot lengths while maximizing the transmission for short slot lengths. From the measured spectra (solid curves in Figure 2(a)), the nanoslots transmit mostly the polarization component perpendicular to the slot direction [55], with transmittance peaks at 684, 747, and 797 nm for slot lengths 145, 172.62, and 190 nm (solid curves, H-pol), respectively. Note that there is still some small residual transmission (dashed curves) along the slot direction. However, it can be treated as a background as it is much smaller than *T*_HH_ and insensitive to *L*. Moreover, the *L* = 145 nm nanoslot array exhibits the largest transmittance ratio ( $\frac{T_{\text{HH}}}{T_{\text{VV}}}∼9.77$ ) at wavelength 684 nm which is chosen as the working wavelength in the sample design and the coincidence imaging measurements. Figure 2(b) shows the transmission amplitude of the nanoslot array at wavelength 684 nm from Figure 2(a) with different *L* for both H and V polarizations. The solid red line is a linear fit ( $\left|t_{\text{HH}}\right|=2.127-0.009\;L$ where *L* is in unit of nm) of the H-pol data and the black dashed line is the mean (0.252 ± 0.004) of the V-pol data. It becomes our design principle in giving different transmittance by varying *L*. From the fitted results, the nanoslot array is expected to give a transmittance ratio from 2.17 to 9.77 when the slot length decreases from 190 nm to 145 nm.

Figure 2(c) shows the experimental setup for the coincidence measurement. The incident beam from a supercontinuum pulse laser is decomposed by a monochromator to give coherent pulses at 684 nm with a 3 nm bandwidth. It is then split into two beams by a polarization beam splitter with one beam passing through a motorized optical delay line. The two beams then recombine after merging at a second polarization beam splitter. A quarter waveplate (QWP), fast axis at 45° from the vertical, behind the second beam splitter converts the two beams into two orthogonal right-handed and left-handed circular polarization (RCP and LCP) incident light for the metasurface sample. A 100 mm lens then focuses the circularly polarized beams with overlapping spot sizes large enough to cover the whole sample. After passing through the sample, the transmitted beams will be collected by a 10× objective, projected by another QWP and analyzer to LCP, and finally collected by the SPAD camera for coincidence measurement. Here, the required phase randomization needed for the two-photon interference with weak coherent states is realized by the vibrations resulting from the mechanical motion of the optical components along the motorized optical delay line [34]. Figure 2(d) shows the measured second-order coherence *g*_2_ plot as a function of optical delay between the reference H and V polarization channels, i.e. $P_{{ii}^{\prime }}/{\left(P_{\text{ref}}^{\left(b\right)}\right)}^{2}$ . Due to the photon bunching effect in the two-photon interference for simultaneously incident pulses, the measured coincidence count is expected to drop by half compared to the case without the interference (large delay), producing coincidence visibility of 0.5 [50–52]. The red solid curve in Figure 2(d) is a Gaussian fit to the data giving coincidence visibility of 0.46 ± 0.02 which is very close to the predicted value confirming the quality of the sample.

#### 3.2 Polarization-response imaging on metasurfaces

**Figure 3:**
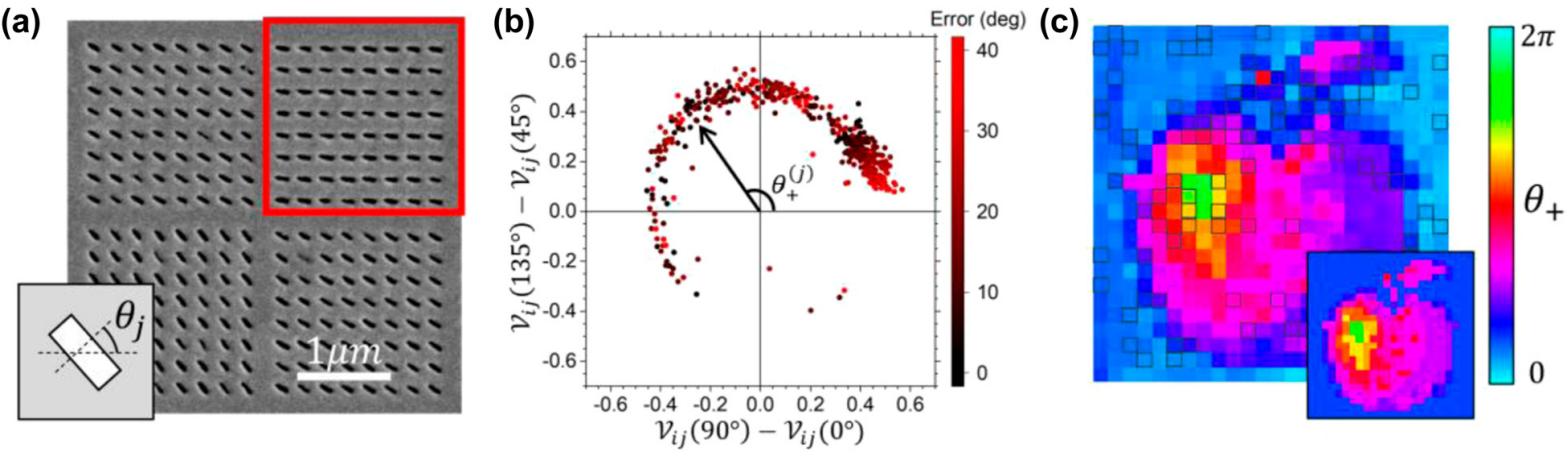
1 degree of freedom coincidence imaging. (a) Zoom-in SEM image of the fabricated sample. Each pixel of the reference or object region (red square box) consists of an array of nano-slots with the same fixed orientations *θ*_*j*_ that control the polarization angle of transmitted light of the pixel. (b) The measured differential visibility between A-pol (*θ*_*i*_ = 135°) and D-pol (*θ*_*i*_ = 45°) plotted against the one for V-pol (*θ*_*i*_ = 90°) and H-pol (*θ*_*i*_ = 0°). The argument of the off-diagonal element of the Jones matrix $\theta_{+}^{\left(j\right)}$ at object pixel *j* can be retrieved from the polar angle. The color of the data point corresponds to the error of retrieved results, taken as the smallest absolute difference between the target and retrieved $\theta_{+}^{\left(j\right)}$ . (c) Retrieved profile $\theta_{+}^{\left(j\right)}$ of the unknown object based on the differential visibilities. The lower-right inset is the target profile. The “noise” due to dead pixels (highlighted in open squares) from the camera is corrected by replacing them with the averaged value of the neighboring pixels.

Figure 3 shows the first example of coincidence imaging on one DOF: argument of the off-diagonal element of the Jones matrix $\theta_{+}^{\left(j\right)}$ in Eq. (4) with |*t*_*j*_| = |*t*_ref_| and $\theta_{-}^{\left(j\right)}=0$ . The object is constructed from only a single kind of nano-slots having the same slot length while the slot orientation *θ*_*j*_ varies in different locations. Figure 3(a) shows the SEM image of the slot arrays. For simplicity, the slot arrays are divided into squares of constant slot orientation with a size of 2 μm × 2 μm (shown within a red square). $\theta_{+}^{\left(j\right)}$ is then related to slot orientations through the Pancharatnam–Berry phase through $\theta_{+}^{\left(j\right)}=2\theta_{j}$ . The whole designed profile is visualized in the inset of Figure 3(c) using a cyclic hue color scheme with cyan color representing value zero. To experimentally extract the $\theta_{+}^{\left(j\right)}$ at object pixel *j*, we consider Eq. (8), the visibility $V_{ij}\left(\theta_{i}\right)$ between the object pixel and the reference panels (different *θ*_*i*_’s), and hence the differential visibilities between the V and H polarization, and between the A and D polarizations are

(10) $$\begin{array}{c} V_{ij}\left(90{}^{\circ}\right)-V_{ij}\left(0{}^{\circ}\right)=\left(1-P_{\text{ref}}^{\left(b\right)}\right)\cos\theta_{+}^{\left(j\right)},\\ V_{ij}\left(135{}^{\circ}\right)-V_{ij}\left(45{}^{\circ}\right)=\left(1-P_{\text{ref}}^{\left(b\right)}\right)\sin\theta_{+}^{\left(j\right)}. \end{array}$$

Figure 3(b) shows the two measured differential visibilities plotted against each other for all the object pixels, roughly falling on a circle of radius smaller than unity when higher order correction (Eq. (8)) of $P_{\text{ref}}^{\left(b\right)}$ is included in Eq. (10). The polar angle subtended by a data point of a particular pixel on this graph is the corresponding measured $\theta_{+}^{\left(j\right)}$ while the color of each data point corresponds to the absolute error of $\theta_{+}^{\left(j\right)}$ between its measured and designed value, with an 18.7° error on average. Figure 3(c) shows the obtained $\theta_{+}^{\left(j\right)}$ profile in comparison with the target profile (inset) showing excellent similarity. The open squares are dead pixels from the SPAD camera that are corrected by replacing them with the averaged value of the neighboring pixels. On the other hand, to improve the signal-to-noise ratio of the image, the coincidence counts between an object pixel and a particular reference panel are averaged over all the reference pixels, 6 in our case, which can be traced back to the same reference panel.

**Figure 4:**
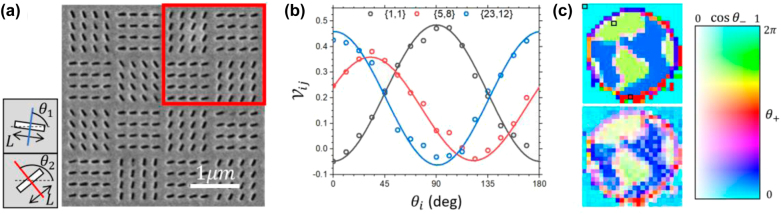
2 degrees of freedom coincidence imaging. (a) Zoom-in SEM image of the fabricated sample. Each pixel in Figure 3(a) (red square) is divided into a checkerboard format with a neighboring sub-pixel consisting of nano-slots with the same length but different slot orientations (*θ*_1_ and *θ*_2_) as indicated by the insets. The red and blue lines represent the slot’s passing axes of the nano-slots. (b) The measured visibility $\left(V_{ij}\right)$ as a function of the reference angle *θ*_*i*_ for three example pixels (indicated in (c) by open squares) of the object. The solid curves are fit using the visibility model. (c) The target image (upper figure) and the retrieved image (lower figure). For better comparison, the images are colored by mapping the values of *θ*_+_ and cos *θ*_−_ to the hue and saturation, respectively, of the HSB color scale for unitary brightness. Note that the dead pixels are smoothed out similar to Figure 3(c).

Next, we perform a 2 DOF coincidence imaging example including the sub-lattice structures. Each slot array of the object (red square) now consists of two sets of slot orientations, *θ*_1_ and *θ*_2_ in a checkerboard pattern, as shown in the SEM image in Figure 4(a). Each slot array gives rise to Jones matrix elements in Eq. (4) where $\theta_{+}^{\left(j\right)}=\theta_{1}^{\left(j\right)}+\theta_{2}^{\left(j\right)}$ and $\theta_{-}^{\left(j\right)}=\left|\theta_{1}^{\left(j\right)}-\theta_{2}^{\left(j\right)}\right|$ . To extract these two parameters, this time we directly fit the visibility $\left(V_{ij}\right)$ with higher order correction of $P_{\text{ref}}^{\left(b\right)}$ (Eq. (8)) as shown in Figure 4(b) for three object pixels against *θ*_*i*_ (labeled in open squares in Figure 4(c)). As the visibility has the form of a cosine function when plotted against *θ*_*i*_, one can extract easily the offset (dip or peak position), amplitude, and mean of the visibility, and hence $\theta_{+}^{\left(j\right)}$ and $\cos\theta_{-}^{\left(j\right)}$ , from the fits (solid curves in Figure 4(b)). Figure 4(c) shows the target image of the Earth (upper figure) and the retrieved image (lower figure) by mapping *θ*_+_ and cos *θ*_−_ to the hue (*h*) and saturation (*s*) of the HSB color scale with unit brightness. The retrieved image appears to be slightly plainer than the target mainly because the visibility function is less sensitive to large $\cos\theta_{-}^{\left(j\right)}$ , which can be shown in Eq. (6) from the term $\cos\theta_{-}^{\left(j\right)}/\left(1+\cos^{2}\theta_{-}^{\left(j\right)}\right)$ .

**Figure 5:**
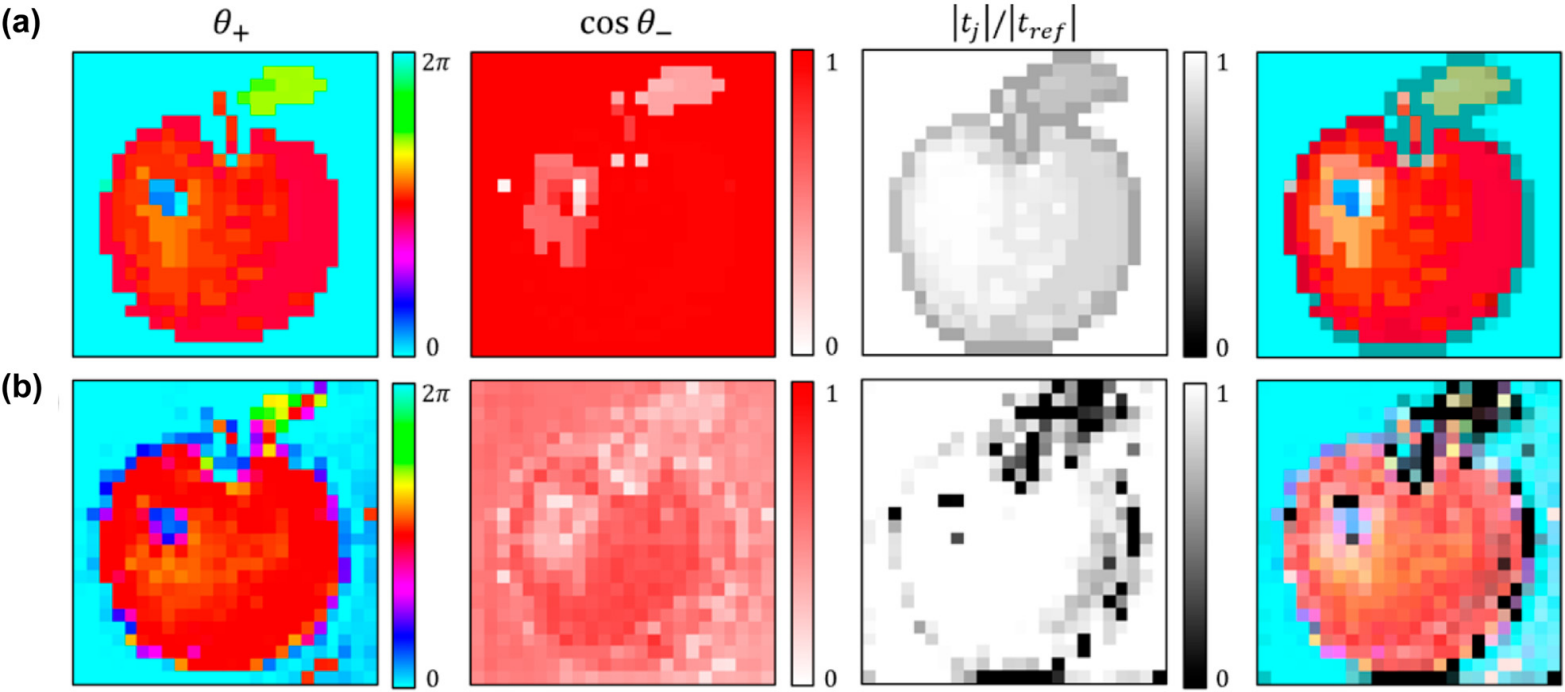
3 degrees of freedom Jones matrix coincidence imaging. (a) The Jones matrix image (right-most image) is obtained by mapping the 3 image profiles in $\theta_{+},\cos\theta_{-},\left|t_{j}\right|/\left|t_{\text{ref}}\right|$ of a target (an apple) into an HSB color image (hue as first column, saturation as second column and brightness as third column as visualization of the Jones matrix elements). (b) The retrieved Jones matrix image profiles of an unknown object. The retrieved profiles in $\theta_{+},\cos\theta_{-},\left|t_{j}\right|/\left|t_{\text{ref}}\right|$ are obtained by fitting the dependence of the object’s visibility (as mentioned in Figure 4), which is then used to reconstruct the object’s Jones matrix profile.

As a final example, Figure 5 shows the 3 DOF coincidence imaging example with additional control on the transmission amplitude $\left|t_{j}\right|$ by varying slot length *L* from 145 nm to 175 nm. As defined in Eq. (4), the object’s Jones matrix at each location is fully characterized by three parameters $\theta_{+},\cos\theta_{-},and\left|t_{j}\right|$ , up to a global phase factor. The measured Jones matrix image can be plotted by mapping all 3 matrix’s DOF to the HSB color profile as shown in the right figure in Figure 5(a) for the object (here an apple). Similar to Figure 4, the profile of $\theta_{+},\cos\theta_{-}and\left|\frac{t_{j}}{t_{\text{ref}}}\right|$ are retrieved from visibility fit with $P_{\text{ref}}^{\left(b\right)}=0.5421$ (from averaged single photon count of the reference panels). The color mapping from the three parameters to the hue (*h*), saturation (*s*), and brightness (b) of the HSB color scale is similar to Figure 4 with $h=\frac{1}{2}+\frac{\theta_{+}}{2\pi }\left(\text{mod}\;1\right)$ , *s* = cos *θ*_−_ and $b=\left|\frac{t_{j}}{t_{\text{ref}}}\right|$ . In comparison to the target profile, the retrieved Jones matrix images show good consistency in terms of the shape of the object and also color representation. While the retrieved profile in *θ*_+_ give excellent consistency, the retrieved profile in cos *θ*_−_ appears to be less accurate since the coincidence visibility is less sensitive to larger cos *θ*_−_, resulting in a paler image similar to Figure 4. For $\left|\frac{t_{j}}{t_{\text{ref}}}\right|$ , the difference in target and retrieved profile mainly comes from the error in estimating the expected $\left|t_{j}\right|$ in an object due to the error in fabricated slot width and also the small array size in the object due to the size limitation.

As mentioned before, conventionally a series of intensity measurements (minimal 10) with different incident polarizations and different polarization analyzers are required to completely determine an unknown 2 × 2 Jones matrix with 7 real number DOF without the global phase [42–44]. For our method, as we have already parallelized different input polarizations using the reference panels, we will only need 3 different cases of final analyzers that reduce the 10 configurations to 3. We expect that by further introducing linear phase gradients (e.g. cascading to a second metasurface) to split the light from the object into different directions for different output polarizations, the number of required experiments can be further suppressed [7, 30]. The freedom in using different designs of meta-atom as the reference panels also allows further generalization of parallelization to other DOF like orbital angular momentum. Compared to Stokes cameras [5–7] which measure the polarization state of scattered light from an object, our approach will be able to measure the response matrix due to the parallelization of input polarizations.

## 4 Conclusions

In conclusion, we have applied two-photon interference to image the Jones matrix profile of an unknown object using metasurface panels with known polarizations as references in parallel. By using two orthogonal circularly polarized phase-randomized weak coherent pulses with tunable delay as inputs, pair-wise coincidence measurements are performed using images taken by a single-photon avalanche diodes (SPAD) camera. The coincidence between each sub-region in the reference channel and all pixels in the object channel are obtained as a series of coincidence images as a function of reference polarization and optical delay. The change in the coincidence visibility at each object’s location/pixel as a function of reference polarization can then be used to retrieve its polarization properties. Conventionally, determining the Jones matrix of the material requires multiple configurational measurements in switching between the different combinations of incidence and analyzing polarizations. On the contrary, for our work, the post-selection of coincidence images with specific reference polarization in our approach eliminates the need in switching the incident polarization. Thus, the characterization of the Jones matrix is efficient through the parallelization of the incident configuration and image analysis, offering great potential in biological and medical imaging applications. Such a coincidence imaging scheme has the potential in simplifying the Jones matrix imaging process. For the future extension, the switching of analyzing polarization in the current work can be further considered if more complicated Jones matrix imaging than the current work is to be performed.

## Supplementary Material

Supplementary Material Details
