# Supplementary material for: Jones-matrix imaging based on two-photon interference
Source: Nanophotonics. 2022 Oct 24;12(3):579–88. doi: 10.1515/nanoph-2022-0499 (PMC11501585; doi:10.1515/nanoph-2022-0499)
Supplement: Supplementary file 1 — Supplementary Material Details [file j_nanoph-2022-0499_suppl.pdf]

Supplementary Material

Jones-matrix imaging based on two-photon interference

Tsz Kit Yung, Hong Liang, Jiawei Xi, Wing Yim Tam*, and Jensen Li*

Department of Physics, The Hong Kong University of Science and Technology, Clear Water Bay, Hong Kong, China

**Correspondence and requests for materials should be addressed to:*

* phtam@ust.hk (W.Y.T.), jensenli@ust.hk (J.L.)

Details in the theory of Coincidence imaging scheme

This section gives the details on how to obtain Eq. (3b) to Eq. (10) in the main text using the coherent state formulation. Consider the photon statistics directly from the output coherent states α_i and α_j. Assume all the camera's detectors have the same detection efficiency η and it does not distinguish photon number (i.e, only differentiate no photon and has photon in the same frame). The total probability of having a photon detected at the pixels i and j are P_i and P_j:

$$P_i = \sum_{N=1}^{\infty} |\langle N_i|\alpha_i\rangle|^2 (1 - (1-\eta)^N) = 1 - e^{-\eta|\alpha_i|^2}, \tag{S1a}$$

$$P_j = \sum_{M=1}^{\infty} |\langle M_j|\alpha_j\rangle|^2 (1 - (1-\eta)^M) = 1 - e^{-\eta|\alpha_j|^2}. \tag{S1b}$$

$|N_i\rangle$ and $|M_j\rangle$ are the Fock state denoting N and M photons at the pixel i and j. $|\alpha_i\rangle$ and $|\alpha_j\rangle$ represents the coherent state with the corresponding complex amplitudes. The coincidence probability P_{ij} between different pixels i and j are given by (as Eq. (3a) in the maintext):

$$P_{ij} = \sum_{N=1}^{\infty}\sum_{M=1}^{\infty} |\langle N_i M_j|\alpha_i \alpha_j\rangle|^2 (1 - (1-\eta)^N)(1 - (1-\eta)^M) = P_i P_j. \tag{S2}$$

The last equal sign in Eq. (S2) comes from the properties of coherent states. From Eq. S1, the single-photon count for pixel i and the ballistic one (superscript "b") is therefore derived using the expression of α_i and α_j given in Eq. (1) in the maintext:

$$P_i = 1 - e^{-\eta|t_{iL}\alpha_L + t_{iR}\alpha_R|^2}, \tag{S3a}$$

$$P_i^{(b)} = 1 - e^{-\eta(|t_{iL}\alpha_L|^2 + |t_{iR}\alpha_R|^2)}, \tag{S3b}$$

and similarly for pixel j:

$$P_j = 1 - e^{-\eta|t_{jL}\alpha_L + t_{jR}\alpha_R|^2}, \tag{S4a}$$

$$P_j^{(b)} = 1 - e^{-\eta(|t_{jL}\alpha_L|^2 + |t_{jR}\alpha_R|^2)}. \tag{S4b}$$

To observe the effect of two-photon interference with coherent states, phase randomization is required by ensemble averaging a uniformly random phase difference ϕ between the two incident beams α_R and α_L (denoted by an angle bracket $\langle\ \rangle$). By using $\frac{1}{2\pi}\int_0^{2\pi} e^{-(|a|+|b|\cos\phi)}\,d\phi = e^{-|a|} I_0(|b|)$, we have:

$$\langle P_{ij}\rangle = P_{ij}^{(b)} - \left(1 - P_i^{(b)}\right)\delta_i - \left(1 - P_j^{(b)}\right)\delta_j + \left(1 - P_i^{(b)}\right)\left(1 - P_j^{(b)}\right)\delta_{ij}, \tag{S5}$$

with $P_{ij}^{(b)} = P_i^{(b)} P_j^{(b)}$, $\delta_i = I_0(2\eta|t_{iL}\alpha_L||t_{iR}\alpha_R|) - 1$, $\delta_j = I_0(2\eta|t_{jL}\alpha_L||t_{jR}\alpha_R|) - 1$ and $\delta_{ij} = I_0\left(2\eta\sqrt{|t_{iR}t_{jL} + t_{jR}t_{iL}|^2|\alpha_R|^2|\alpha_L|^2 + \left(|t_{iR}\alpha_R|^2 - |t_{jR}\alpha_R|^2\right)\left(|t_{iL}\alpha_L|^2 - |t_{jL}\alpha_L|^2\right)}\right) - 1$ for the zero-order Bessel function I_0. At small η, expanding Eq. (S3b), (S4b), and (S5) with $|\alpha_L| = |\alpha_R|$ up to the lowest order of η give:

$$P_i^{(b)} \cong \eta(|t_{iL}\alpha_L|^2 + |t_{iR}\alpha_R|^2) \triangleq P_{\text{ref}}^{(b)}, \tag{S6a}$$

$$P_j^{(b)} \cong \eta\left(|t_{jL}\alpha_L|^2 + |t_{jR}\alpha_R|^2\right), \tag{S6b}$$

$$\langle P_{ij}\rangle \cong \left(P_{\text{ref}}^{(b)}\right)^2\left(\frac{|t_{jL}|^2 + |t_{jR}|^2}{|t_{iL}|^2 + |t_{iR}|^2} + \frac{2Re\left(t_{iL}t_{iR}^* t_{jL}^* t_{jR}\right)}{(|t_{iL}|^2 + |t_{iR}|^2)^2}\right). \tag{S6c}$$

In Eq. (S6a), as $P_i^{(b)}$ is a known constant for all the reference pixel i, the reduced form of $P_i^{(b)}$ is defined as $P_{\text{ref}}^{(b)}$ in the maintext with Eq. (S6c) corresponding to Eq. (3b). The following equation, Eq. (5) in the maintext, can be obtained by substituting the expression of t_{iL}, t_{iR} and t_{jL}, t_{jR} into Eq. (3b). From Eq. (2) and (4), $t_{iL} = t_{\text{ref}}, t_{iR} = t_{\text{ref}}\, e^{-2i\theta_i}$, $t_{jL} = t_j$ and $t_{jR} = t_j \cos\theta_-^{(j)}\, e^{-i\theta_+^{(j)}}$, which gives:

$$\langle P_{ij}\rangle \cong \frac{1}{2}\left(P_{\text{ref}}^{(b)}\right)^2\left|\frac{t_j}{t_{\text{ref}}}\right|^2\left(1 + \cos^2\theta_-^{(j)} + \cos\theta_-^{(j)}\cos\left(2\theta_i - \theta_+^{(j)}\right)\right), \tag{S7}$$

and hence the visibility on coincidence count with phase randomization V_{ij} can be derived using Eq. (S6) and its definition:

$$V_{ij} \triangleq 1 - \langle P_{ij}\rangle / \left(P_{\text{ref}}^{(b)} P_j^{(b)}\right) \cong -\frac{\cos\theta_-^{(j)}}{1 + \cos^2\theta_-^{(j)}}\cos\left(2\theta_i - \theta_+^{(j)}\right), \tag{S8}$$

with the ballistic single photon count rate for the object pixel written as

$$P_j^{(b)} \cong \frac{1}{2} P_{\text{ref}}^{(b)} \left|t_j/t_{\text{ref}}\right|^2 (1 + \cos^2 \theta_-^{(j)}). \tag{S9}$$

In our experiment, to obtain the higher-order contribution of $P_{\text{ref}}^{(b)}$ in the visibility V_{ij}, the full formula of $\langle P_{ij} \rangle$ and $P_{ij}^{(b)}$ are used when calculating V_{ij}, which is then expanded to obtain the first-order term in η in comparison to Eq. (S8), which gives Eq.(S10) (Eq. (8) to Eq. (10) in the maintext):

$$\mathcal{V}_{ij}(\theta_i) \cong \beta + \gamma \cos\left(2\theta_i - \theta_+^{(j)}\right), \tag{S10a}$$

where

$$\beta \cong \frac{1}{4} P_{\text{ref}}^{(b)} \left(1 + \frac{2\cos^2 \theta_-^{(j)}}{1 + \cos^2 \theta_-^{(j)}} \left|t_j/t_{\text{ref}}\right|^2\right), \tag{S10b}$$

and

$$\gamma \cong \left(1 - \frac{1}{4} P_{\text{ref}}^{(b)} \left(2 + \left(1 + \cos^2 \theta_-^{(j)}\right)\left|t_j/t_{\text{ref}}\right|^2\right)\right) \frac{-\cos \theta_-^{(j)}}{1 + \cos^2 \theta_-^{(j)}}. \tag{S10c}$$

In the simplified case where the visibility contains only one unknown parameter $\theta_+^{(j)}$ in Fig.3 with $|t_j| = |t_{\text{ref}}|$ and $\theta_-^{(j)} = 0$, the differential visibility between the V and H polarization, and between the A and D polarizations can be obtained from Eq. (S10a):

$$\begin{aligned} \mathcal{V}_{ij}(90°) - \mathcal{V}_{ij}(0°) &= (1 - P_{\text{ref}}^{(b)}) \cos \theta_+^{(j)}, \\ \mathcal{V}_{ij}(135°) - \mathcal{V}_{ij}(45°) &= (1 - P_{\text{ref}}^{(b)}) \sin \theta_+^{(j)}. \end{aligned} \tag{S11}$$
